# Advances and Challenges in Fluorescence *in situ* Hybridization for Visualizing Fungal Endobacteria

**DOI:** 10.3389/fmicb.2022.892227

**Published:** 2022-05-26

**Authors:** Demosthenes P. Morales, Aaron J. Robinson, Andrew C. Pawlowski, Caitlyn Ark, Julia M. Kelliher, Pilar Junier, James H. Werner, Patrick S. G. Chain

**Affiliations:** ^1^Center of Integrated Nanotechnologies, Los Alamos National Laboratory, Los Alamos, NM, United States; ^2^Biosecurity and Public Health, Los Alamos National Laboratory, Los Alamos, NM, United States; ^3^Department of Genetics, Harvard Medical School, Boston, MA, United States; ^4^Wyss Institute for Biologically Inspired Engineering, Harvard University, Boston, MA, United States; ^5^Institute of Biology, University of Neuchâtel, Neuchâtel, Switzerland

**Keywords:** fungi, fluorescence *in situ* hybridization, endobacteria, symbiosis, bioimaging

## Abstract

Several bacteria have long been known to interact intimately with fungi, but molecular approaches have only recently uncovered how cosmopolitan these interactions are in nature. Currently, bacterial–fungal interactions (BFI) are inferred based on patterns of co-occurrence in amplicon sequencing investigations. However, determining the nature of these interactions, whether the bacteria are internally or externally associated, remains a grand challenge in BFI research. Fluorescence *in situ* hybridization (FISH) is a robust method that targets unique sequences of interest which can be employed for visualizing intra-hyphal targets, such as mitochondrial organelles or, as in this study, bacteria. We evaluate the challenges and employable strategies to resolve intra-hyphal BFI to address pertinent criteria in BFI research, such as culturing media, spatial distribution of bacteria, and abundance of bacterial 16S rRNA copies for fluorescent labeling. While these experimental factors influence labeling and detection of endobacteria, we demonstrate how to overcome these challenges thorough permeabilization, appropriate media choice, and targeted amplification using hybridization chain reaction FISH. Such microscopy imaging approaches can now be utilized by the broader research community to complement sequence-based investigations and provide more conclusive evidence on the nature of specific bacterial–fungal relationships.

## Introduction

Fungi and bacteria coexist in complex and diverse environments, ranging from soil to within the digestive systems of humans and other mammals (Huttenhower et al., 2012; Deveau et al., 2018). However, only recently have we begun to uncover the breadth of interactions between these kingdoms. Bacterial–fungal interactions (BFI) can be diverse in nature, spanning from associations which occur at the hyphal surface to associations where fungi harbor bacteria with obligate endohyphal lifestyles. These close associations can also alter the physiology of either or both participants, with previously observed impacts on fungal reproduction, secondary metabolite production, and bacterial genome evolution (Vahdatzadeh et al., 2015; Mondo et al., 2017; Schulz-Bohm et al., 2017; Uehling et al., 2017). Yet, a common challenge in the emerging field of BFI research is how to distinguish between transient and more enduring interactions.

Many studies have found co-occurrence patterns between fungi and bacteria. One of the most commonly used methods for studying microbial communities is to amplify 16S rRNA gene sequences from fungal isolates, which otherwise appear axenic. While this method can help assess the diversity of potential bacterial associates within a particular fungal host, it does not allow for further spatial characterization of these associates, such as determining the load or spatial distribution of such symbionts on or within fungal isolates. Thus, microscopy-based investigations are a complementary necessity to sequence-based approaches, as they allow for a more complete understanding of the spatiotemporal nature of BFI. In particular, transmission electron microscopy provides high-resolution structural information of internal fungal components and has been used to visualize bacterial associates of fungal cultures (Desirò et al., 2014; Uehling et al., 2017). However, the time-consuming sample preparation and limited access to specialized electron microscopes make it challenging to perform exploratory investigations. Moreover, as the contrast in electron microscopy comes from differences in electron density, this method is limited in labeling strategies. Alternatively, optical imaging techniques, such as fluorescent *in situ* hybridization (FISH), afford improved spatial context of interactions by providing a wider field of view and enabling target specificity. As such, FISH remains a minimal requirement for *in situ* targeted-sequence analysis (Higuchi and Takegawa, 2020; Bartholomai et al., 2021) and can be a valuable tool for assessing spatiotemporal aspects of bacteria with fungal hypha.

Fluorescence *in situ* hybridization (FISH) is a versatile fluorescence imaging technique that employs targeted nucleic acid probes to label cellular DNA, RNA, or ribosomal sequences. The specificity of the targeted probes makes FISH the foremost tool for visualizing the taxonomic distributions of organisms, such as bacteria, archaea, and eukaryotes within microbiomes at small spatial scales (Amann et al., 1990b; Kobabe et al., 2004; Zhou et al., 2007; Yamaguchi et al., 2015; Hatzenpichler et al., 2016). Imaging with FISH has been performed on a diversity of biological tissues and samples, but in fungi, this technique has been most frequently performed on early diverging fungi from Mucoromycota (Okrasińska et al., 2021). This is in part due to the fact that the Mucoromycota are widely studied because of their propensity to form mycorrhizal associations with plants, but also because these fungi contain a number of taxa that have been shown to host endosymbiotic bacteria making them ideal to serve as model BFI systems. We now realize that endosymbiosis and interactions between fungi and bacteria are more widespread and occur across a vast and diverse species of bacteria and fungi (Robinson et al., 2021). The results from this large survey have increased the desire to utilize established methods, such as FISH, on diverse fungal lineages to further investigate and characterize the microbiomes of fungi.

In this study, partly in order to support and complement sequence-based analysis of the presence of bacteria interacting with fungi, we sought to address certain challenges in *in situ* hybridization methods and optimize sample preparation methods for the visualization of spatially distinct bacterial cells within fungal hyphae. We focus on endobacterial associates because access to well-studied, tractable model interactions are available and greatly facilities investigations into how to address these challenges and optimizations. We examined several parameters that may affect access of FISH staining to intracellular bacteria, such as mycelial location, cell wall permeability, and media conditioning. Isolates from the fungal family Mortierellaceae, including *Mortierella*, *Podila*, and *Linnemannia* which harbor *Mycoavidus* as a bacterial endosymbiont were used as the primary model for comparisons in this work (Uehling et al., 2017). Selection of this model was based on the knowledge that this particular association is highly tractable and the bacterial endosymbiont is predicted to be present at high densities, making it advantageous for microscopy-based investigations. Furthermore, while interactions between Mortierellaceae and *Mycoavidus* are widely studied, spatiotemporal aspects of these associations remain largely unexplored. We also investigated alternatives to standard FISH imaging and the feasibility of fluorescence amplification techniques for the detection of bacterial associates of fungi that are found at lower abundance or with lower 16S rRNA copy numbers than with *Mycoavidus*. The results of this work demonstrate that preparing fungal isolates for imaging experiments with FISH is non-trivial, and optimization of fluorescence signal-to-noise ratio (SNR) requires unique approaches that are dependent on the specific fungus of interest.

## Materials and Methods

### Media Discs

The following medias were prepared at 1× liquid solutions as per manufacturer’s instruction: malt extract broth (ME, BD Difco), potato dextrose broth (PD, BD Difco), Reasoner’s 2A broth (R2A, Teknova), Vogel’s minimal media (VM, Fungal Genetics Stock Center). Thin solid media discs were prepared by combining powdered agar (BD Bacto Agar, Thermo Fisher Scientific) or Phytagel (gellan gum, Sigma-Aldrich) slowly to a liquid solution of media broth to a final concentration of 2% and 4%, respectively. Hydrogel slide preparation was adapted from methods described by Woo et al. (2010). The solutions were autoclaved and the molten media were cast between two 70% ethanol sterilized standard microscope slides (VWR) separated by two 24 × 60 mm No. 1.5 coverglass (Corning) and set to solidify for at least 10 min under sterile conditions in a biosafety cabinet. The microscope slides were carefully separated with a razor and the coverglass was removed. The mouth of a sterile 15 ml Falcon conical tube (Fisher Scientific) was used to cut ~17 mm diameter circular discs from the cast media. For agar discs, excess media was removed from the slide and the pads were gently slid across to an adjacent clean new slide. For Phytagel discs, a razor was used to gently lift the disc and with a sterile inoculation loop (VWR) the disc was transferred a new glass slide. Slides were then fitted with a FastWell reagent barrier (Grace Bio-Labs). The glass slides were placed in a 100 mm Petri dish with a water-saturated Kim Wipe to maintain humidity. For disc autofluorescence detection, 4′,6-diamidino-2-phenylindole (DAPI, Thermo Fisher Scientific) and acridine orange (Sigma-Aldrich) were added at 1 and 10 μg•ml^−1^, to the discs, respectively, and incubated for 10 min before washing three times with 2× saline-sodium citrate (SSC, Thermo Fisher Scientific).

### Fungal Culture

Mortierellaceae isolates were derived from a collection of environmental isolations, predominantly soil-based studies, and have been maintained in culture for several years. These cultures from our collection are denoted with the reference identification numbers beginning with “LANL.1351.” The isolates are denoted as follows: *Podila verticillata* = (*Mortierella verticillata*; LANL.1351.123, NCBI BioSample: SAMN19716290; Vandepol et al., 2020), and three *Mortierella alpina* (LANL.1351.60, NCBI BioSample: SAMN19716328; LANL.1351.61, NCBI Bio Sample: SAMN19716329; LANL.1351.62, NCBI BioSample: SAMN19716330). Mucoromycota endobacterial positive and negative isolates were acquired from ATCC, *Rhizopus* spp. (cat. no. 20577) and *Rhizopus oryzae* (cat. no. 24794), respectively. The fungi were grown on 100 mm Petri dishes of 2% bacto agar (VWR) supplemented with 1× broth of the following media: ME, PD, R2A, and VM. Cultures were incubated for 5+ days at 25°C and a <1 mm^2^ dissection was cut from the mycelial mat and transferred to the center of a media disc corresponding to the appropriate media. Cultures were further grown for 3–5 days to spread mycelium across the disc. For hybridization chain reaction, ~1–2 mm^2^ mycelia dissections were taken directly from fungal cultures and transferred to a 1.5 ml microcentrifuge tube (Eppendorf) for staining.

### Fungal Genome Sequencing and Assembly

Genomic DNA was extracted from a pure culture of LANL.1351.123 grown in malt extract broth using the FastDNA™ SPIN kit for Soil (MP Biomedicals). Genome sequencing was performed using the Illumina NextSeq platform configured for paired-end 151 base pair reads. A total yield of 23 Gbp were obtained for this sample. The FaQCs^1^ module in EDGE^2^ was used with default settings for pre-processing and quality control of sequences. Reads which passed quality control (~541x genome coverage) were assembled using SPAdes v3.15.4.^3^ This Whole Genome Shotgun project has been deposited at DDBJ/ENA/GenBank (accession JALPZK000000000).

### Probe Design

Sequencing of the fungal host at a high depth resulted in the additional ability to capture the genome from the bacterial endosymbiont. Barrnap^4^ was used to identify ribosomal RNA genes among the assembled contigs. Small subunit (SSU) rRNA sequences for both the fungal host (NCBI accession ON357719) and the bacterial endosymbiont (ON357872) were extracted for use in probe design. Taxonomic-specific FISH probes were designed using Python code BioPython package adapted from Liu et al. (2021). In summary, the script was written for the specific purpose of parsing both the 16S rRNA sequence from the bacterial endosymbiont and the 18S rRNA sequence from the fungal host. Additionally, all other assembled contigs (excluding contigs containing rRNA regions) obtained from sequencing of the fungal host were included to ensure specificity. Each sequence was divided into every possible 25-mer, and unique 25-mers were isolated by comparing sets. Considerations for target selection included checking the melting temperature against the temperature required for both the hybridization and ligation steps and using the Levenshtein distance module to search for indels within the target to make off-target hybridization unlikely. This process included searching the last 18-nucleotide (nt) segments of the 25-mers and selecting only target sites where the Levenshtein distance was greater than 6 compared with all non-self 18-nt segments. Probe assembly consisted of taking the reverse complement of each unique target RNA. Then, each potential probe was searched to avoid self-complementary 4-mers and G-quadruplex structures, yielding final probe selection. A link to the custom Python scripts used for probe design are provided on GitHub: https://github.com/dmorales003/endobacteria_analysis. The final 16S rRNA probe for *Mycoavidus* used in this study was 5′-ACGTCATCCCC GCCTTCCTCCGGTT-3′ and 18S rRNA for *P. verticillata* 5′-CCATACTCCCCCCGGAACCCAAAAA-3′. After probes were designed, they were tested for specificity by aligning to the genome assembly obtained from LANL.1351.123 (JALPZK000000000) using blastn (−task blastn-short).^5^ The only significant alignments found in the blastn search were the contigs containing the 16S and 18S rRNA sequences used to design the probes, indicating a low probability of off-targeting within the fungal sample. From our own microscope system, we found that unstained, fixed fungal mycelia exhibited relatively higher autofluorescence under blue light excitation, which was also observed in several fungi in previous reports (Knaus et al., 2013). Therefore, to maximize SNR we labeled the 16S rRNA and 18S rRNA probes on the 5′ end with Cy3 and AlexaFluor 647, respectively.

### Fixation and Cell Wall Lysis

Fixation in formaldehyde was adapted from the protocols described by Bertaux and Wang (Bertaux et al., 2003; Wang et al., 2017). Fungal cultures grown under varied conditions were fixed in a 4% solution of formaldehyde (w/v, Thermo Fisher Scientific) in PBS overnight at 4°C. Fungal biomass was washed 3× with PBS and then treated with cocktail combinations of cell wall digestive enzymes: 1 mg•ml^−1^ solution of lysozyme, or 0.5 mg•ml^−1^ solution of chitinase, and/or 5 mg•ml^−1^ solution of glucanase (reagents purchased from Sigma-Aldrich) for 1 h at 37°C and washed 3× with PBS. The cultures were then dehydrated with a step-wise series of ethanol treatments: 50%, 75%, 100%, 75%, 50%, and finally replaced with PBS for 3 min between each step.

### Standard Fluorescence *in situ* Hybridization

The probe staining procedure was adapted from methods described by Liu et al. (2021). Cell wall digested cultures were pre-treated with a 6.5× solution of SSC supplemented with 0.1 U•ml^−1^ SUPERase RNase inhibitor (Thermo Fisher Scientific) for 30 min at room temperature. The pre-treatment was then replaced with a probe hybridization solution of 6.5× SSC, 15% formamide (Thermo Fisher Scientific), and 125 nM of each probe (*Mycoavidus* 16S rRNA, *P. verticillata* 18S rRNA). The cultures were incubated overnight at 37°C and then washed 5× with 6.5× SSC + 15% formamide for 5 min each at room temperature. We note that only a single putative bacterial associate was observed in the sequence alignment of the probe against the fungal genome. Given this observation, concerns of non-target binding were minimal. Hybridization and probe washing steps were performed at a lower stringency than previously reported methods for environmental microbial samples (Cardinale et al., 2008). The motivation behind this decision was based on previous work which suggested this alteration may aid in maximizing fluorescent signal intensity (Wright et al., 2014). The solution was replaced with 1 μg•ml^−1^ DAPI in 6.5× SSC for 10 min at room temperature. The cultures were washed once more with 5× SSC and finally resuspended in 2× SSC. The discs were then transferred to a microscope slide with an inoculating loop and cast in ProLong Glass antifade mountant (Thermo Fisher Scientific) with a No. 1.5 coverglass. Slides were then placed in a drawer shielded from light for >36 h at room temperature to induce optimal curing according to manufacturer’s protocol.

### Hybridization Chain Reaction and Standard Hybridization Comparison

HCR-FISH staining protocol used in this study was developed by Choi et al. (2014, 2018) and uses probes and reagents purchased from Molecular Instruments. A ~ 1 mm^2^ area of mycelia was gently extracted from the surface of agar plates with an inoculation loop and transferred to a 1.5 ml tube. Fixation and digestion were performed as previously described with chitinase and glucanase. After ethanol treatment, cell wall digested mycelia were pre-treated with hybridization buffer (Molecular Instruments) for 30 min at room temperature and then replaced with hybridization buffer containing 125 nM of HCR initiator probe set based on universal Eub338 region of the 16S rRNA sequence (5′-ACACUGGAACUGAGACACGGUCCAGACUCCUACGGGAGGCAGCAGUGGG-GAA-3′) available from Molecular Instruments. The probe set was designed with the B4 initiator sequence as designed by the manufacturer. For standard hybridization comparison, fungi were stained with the Eub338 probe sequence 5′-GCTGCCTCCCGTAGGAGT-3′ (Amann et al., 1990a), with a Cy3 dye labeling the 5′ end. The initiator was incubated overnight at 37°C and washed 4× with probe wash buffer (Molecular Instruments) at 5 min each at 37°C. The cultures were then conditioned in an amplification buffer (Molecular Instruments), while the AlexaFluor 647-labeled B4-initiator targeting hairpin probe set (Molecular Instruments) were snap cooled as per manufacturer’s protocol. The pre-treated cultures were then incubated with the amplifying probes at a final concentration of 18 pmol in 300 μl amplification buffer (Molecular Instruments) overnight at room temperature. The cultures were washed in 5× SSC three times and treated with 1 μg•ml^−1^ DAPI in 2× SSC for 10 min at room temperature and washed once more with 2× SSC. The mycelia were transferred to a microscope slide spotted with 2× SSC and were imaged immediately after.

### Estimating Endobacterial Burdens Using Quantitative PCR

Fungal cultures were grown on PD agar topped with a nitrocellulose film. Mycelia were collected in a 2 ml tube and DNA was collected using the FastDNA spin kit for soil (MP Biomedicals) according to manufacturer’s protocol. To quantify relative bacterial abundance in the fungal samples the following primers were used to amplify a region of the 16S rRNA gene sequence of the endobacteria: Forward (Eub 338; Lane, 1991): 5′CTCCTACGGGAGGCAGCACT-3′; Reverse (Eub 513; Muyzer et al., 1993): 5′ATTACCGCGG CTGCTGG 3′. DNA and primers were suspended in Sybr Green Master Mix (BioRad) and qPCR was performed using a CFX Connect Real-Time PCR Thermocycler (BioRad). A calibration curve was generated by amplifying a serial dilution of DNA extracted from *Burkholderia thailandensis* E264 lab standard to establish relative comparison of sample copy numbers.

### Amplification and Analysis of 16S rRNA Genes From Fungal Isolates

16S amplicon sequencing was performed in a previous publication (Robinson et al., 2021) and SRAs for the samples are provided in the BioSample accessions above. Briefly, the V3-V4 region of the 16S rRNA gene from fungal genome extractions as described above was amplified by nested PCR. First, primers 27F (Lane, 1991): 5′-AGAGTTTGATYMTGGCTCAG-3′ and 907R (Muyzer and Smalla, 1998): 5′-CCGTCAATTCMTTT GAGTTT-3′ were used to amplify the V1-V5 region of the 16S rRNA gene. Primers 341F (Muyzer et al., 1993): 5′-CCTACGGGNGGCWGCAG-3′ and 806R (Caporaso et al., 2011): 5′-GGACTACHVGGGTATCTAATCC-3′ were then used as an additional step to further amplify the V3–V4 region before being sequenced on Illumina MiSeq (2 × 300 bp paired-end reads). Raw sequences were analyzed using QIIME2 (Bolyen et al., 2019) with DADA2 (Callahan et al., 2016) to produce the amplified sequence variants. The ASVs were then taxonomically classified using QIIME2’s Naïve Bayesian classifier trained on a custom database of the SILVA reference 16S rRNA sequence collection (SSU 138 Ref NR 99) trimmed to the same V3–V4 region produced by the sequencing primers used.

### Fluorescence Microscopy Imaging

Confocal microscopy imaging of fungal samples was performed on an Olympus FV3000 laser scanning confocal microscope using a × 100 oil objective lens NA 1.45 and equipped with 405, 488, 561, and 640 nm excitation laser sources and 405/488 and 561/640 dichroic mirrors. Fluorescence image stitching was performed on a Zeiss Axio Observer microscope using a x100 oil objective lens NA 1.3 and filter for DAPI, Alexa Fluor 488, Cy3, and Cy5. A minimum of five images per sample at locations distributed across the fungal mycelia were collected to acquire a representation of each condition described.

### Image Analysis

Images were kept close to raw as much as possible and were analyzed by automated methods for standardization. Briefly, image stacks from confocal microscope images were arranged as a max intensity Z projection using FIJI (Schindelin et al., 2012). Fluorescence image intensity comparisons were performed on a custom Python script utilizing the Numpy and Matplotlib packages to acquire the minimum and maximum pixel intensity for each image set and normalize the images to the min and max scale. Bacterial cell counting was performed on a custom Python script which can be found at https://github.com/dmorales003/endobacteria_analysis. Using the OpenCV package an adaptive image threshold was performed to generate a mask of the fungal mycelial boundaries on the 18S rRNA images. The 16S rRNA images were masked through the Scikit-image package and bacterial cells were counted using the difference of Gaussian blob detection algorithm. Two-sided hypothesis testing was performed using Welch’s *t*-test using statistics functions provided by the SciPy package. Fluorescence image stitching was performed using pairwise stitching of images through the Stitching plugin in FIJI.

## Results

### Growth on Solid Media Discs

**Figure 1:**
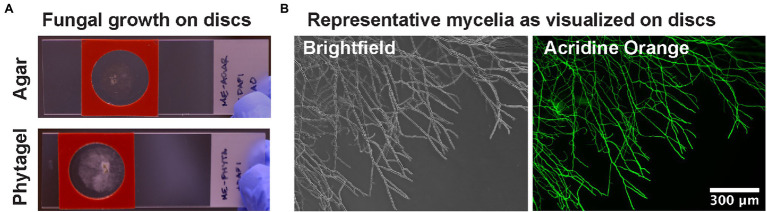
Hydrogel discs for culturing filamentous fungi on slides. **(A)** Discs are molded to be approximately the thickness of a glass coverslip and are suitable for culturing with a reagent barrier for solution exchange in FISH staining methods. **(B)** Example brightfield and fluorescence microscopy images of fungal mycelia grown on hydrogels.

Microscope imaging of fungi can be challenging due to the thick multicellular layers of mycelial mats and the movement of hyphae when unsecured to a surface. Agar blocks are one established method to grow or smear fungi on microscope slides (Woo et al., 2010). However, block thickness affects optical transparency and their relatively large volume is not very amenable with the costly staining reagents used in FISH microscopy experiments. In regards to challenges with opacity, previous reports have examined the optical clarity of several hydrogel substrates for microbial experiments and have determined that Phytagel (gellan gum) presented superior performance in minimizing autofluorescence (Jaeger et al., 2015). We tested that the Phytagel substrate would provide adequate SNR for imaging bacteria within fungi. Fungi were grown on dissolved media in either 2% (w/v) agar or 4% (w/v) Phytagel hydrogels. Mycelia were observed to exhibit procumbent growth on the media surface with limited aerial hyphae formation (Figure 1A). This helped keep wider areas of mycelia in focus across fields of view and allowed for individual hyphae to be resolved (Figure 1B). In regards to autofluorescence of the hydrogels, DAPI and acridine orange stains were added to the slides and washed with PBS to measure residual fluorescence. In support of Jaeger et al. (2015), the Phytagel hydrogels exhibited reduced autofluorescence across the DAPI, FAM, and Cy3 fluorescence channels when compared with the agar hydrogels (Supplementary Figure 1). Furthermore, the 4% Phytagel also promoted greater hydrogel strength, facilitating handling, and consistency in sample preparation (data not shown). All together, these results indicate the Phytagel hydrogel has several advantages for fungal imaging when compared to agar hydrogels.

### Cell Wall Digestion and Permeability

**Figure 2:**
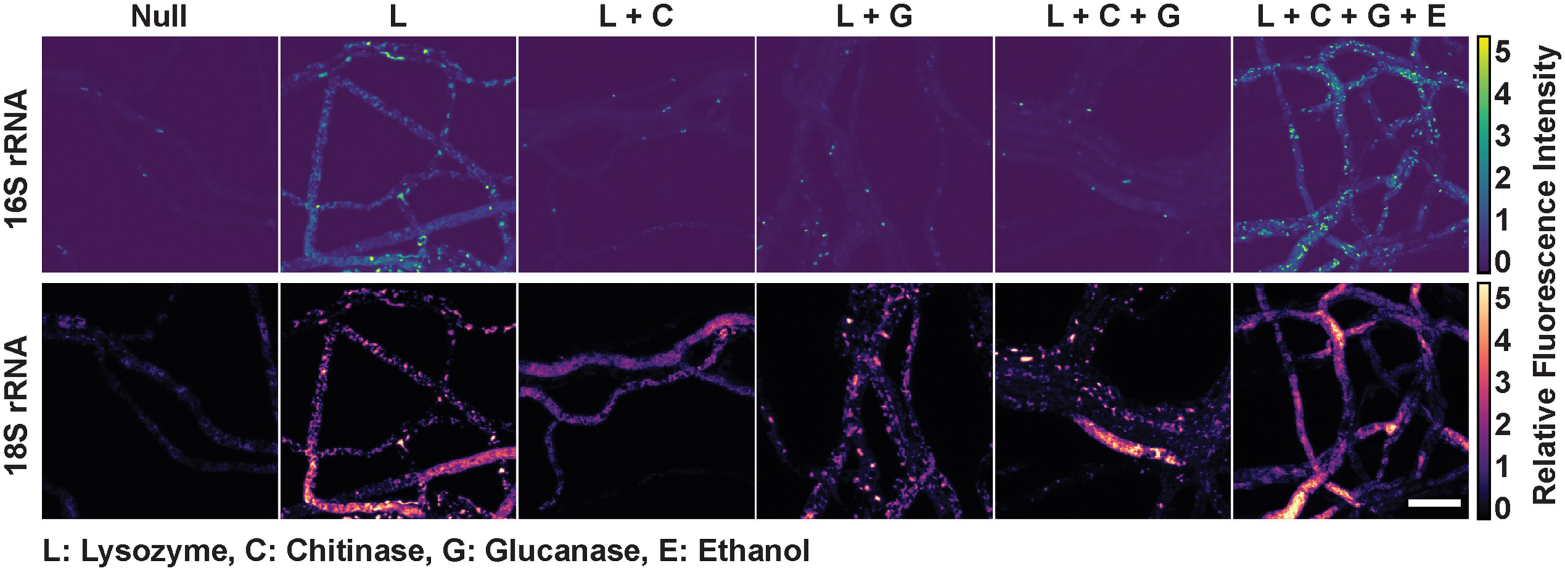
*Mycoavidus* bacterium observed in *Podila verticillata*, LANL.1351.123, by rRNA FISH imaging after exposure to different permeabilization treatments. Relative fluorescence intensity images are scaled to the minimum and maximum pixel intensities of image sets (across) for 16S rRNA or 18S rRNA staining (top and bottom, respectively). Chemical or cell wall digestion enzymes treatments are designated by the single letter codes with key provided. Scale bar is 10 μm.

It has previously been found that enzymes used in lysis of fungal cell walls are not universally effective (Bartholomai et al., 2021). The primary reason for this inconsistent effectiveness is the variability in fungal cell wall composition. Fungal cell walls can contain different amounts of glucans, glycoproteins, chitin, and chitosan. In this study, a set of *P. verticillata* isolate (LANL.1351.123) harboring *Mycoavidus* were grown on slides to determine whether permeabilization of fungal cell walls *via* chemical and enzymatic digestion could influence probe diffusion and intra-hyphal localization examine. Fixed fungal isolates grown on Reasoner’s 2A media (R2A) were incubated with either a solution of lysozyme for bacterial cell wall digestion, fungal cell wall lytic enzymes glucanase or chitinase, or a combination of enzymes with or without chemical permeabilization by ethanol. The untreated control, only fixed by formaldehyde, exhibited minor staining of the endobacterial 16S rRNA as well as fungal 18S rRNA suggesting minor porosity of the fungi after fixation allowing superficial staining of cell objects (Figure 2). Lysozyme treatment of the fungus identified some bacterial morphology but was accompanied by significant autofluorescence in each channel, possibly due to the non-specific binding of lysozyme to nucleic acids (Ghosh et al., 2013). Treatment with either glucanase or chitinase on the other hand improved resolution and SNR of bacterial structures, as well as increased homogeneity of 18S rRNA staining. SNR of bacterial features and 18S rRNA improved when the lysozyme, glucanase, and chitinase were combined. Treatment with ethanol has also been observed to aid in permeabilization by disrupting membranes, extracting lipids, and denaturing proteins (Bartholomai et al., 2021). When the fungal isolate was treated with the addition of ethanol, the SNR of bacterial 16S rRNA was further improved and we observed an increase in 18S rRNA signal homogeneity. Several images were collected per treatment exhibiting similar results. Additional, wider field images also confirm that this effect occurred on the entire *P. verticillata* subculture and not isolated to particular mycelial regions (Supplementary Figure 2). Generalist probe staining is also shown in Supplementary Figure 3. We determined that combining enzymes and ethanol treatments promoted the best staining conditions for this isolate, and was performed for subsequent experiments.

### Culture Media Affects Fungal Translational Activity and Endobacterial Growth

**Figure 3:**
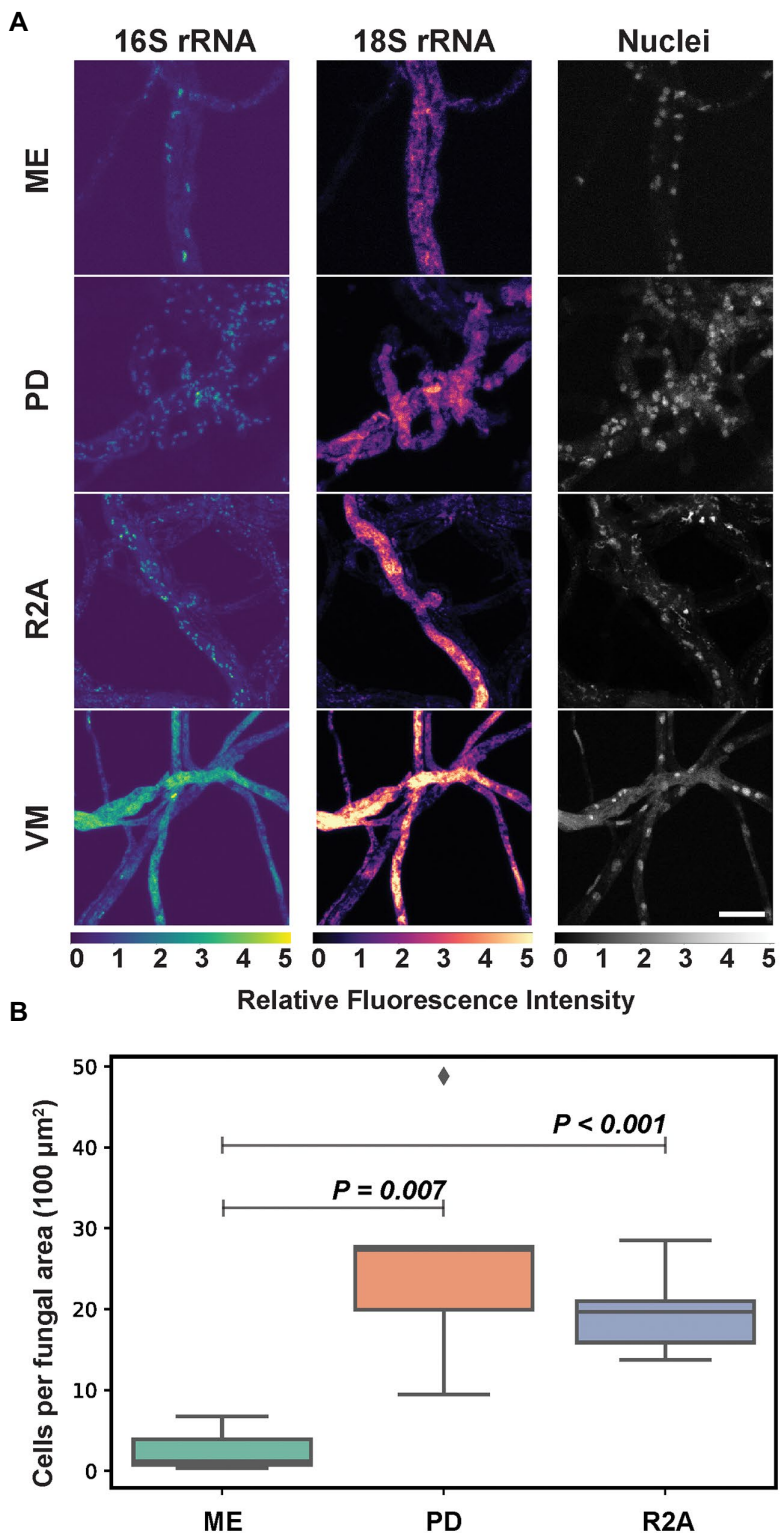
**(A)** *Mycoavidus* (16S rRNA) observed in *P. verticillata* (18S rRNA, LANL.1351.123) grown on Phytagel hydrogel discs supplemented with either Malt Extract (ME), Potato Dextrose (PD), Reasoner’s 2A (R2A), or Vogel’s minimal media (VM). Relative fluorescence intensity images are scaled to the minimum and maximum pixel intensities of image sets (top to bottom) for 16S rRNA, 18S rRNA, or nuclei staining. VM exhibits autofluorescence across all channels under the conditions presented. Scale bar is 10 μm. **(B)** Average *Mycoavidus* bacteria counted across five images of aggregated fungal area of 100 μm^2^ for each media type tested. ME exhibited the significantly least number of bacteria per area compared to PD (*p* = 0.007) and R2A (*p* < 0.001). VM autofluorescence obfuscated bacterial count and was omitted from quantification analysis.

Mycelial morphology and growth phenotypes of fungal isolates can be variably influenced by the composition of culture growth media. Previous work demonstrated that culture media composition can impact the formation of developmental structures in the fungal host, Moura et al. (2020), Sugimoto and Oda (2021) and that these changes coupled with predicted metabolic changes in the fungus could alter endobacterial phenotypes, abundance, and behavior. To determine the effects of culture media composition on endobacterial phenotypes, *P. verticillata* was grown on four different solid media: (R2A) media which is favorable for bacterial growth, Vogel’s minimal (VM) media for fungi, and two common fungal media, potato dextrose (PD), and malt extract (ME) media which provides a slightly more acidic media environment. Color intensities of FISH-labeled 18S rRNA were scaled according to the minimum and maximum pixel values detected to compare the relative intensities of 18S ribosomal targets between conditions. Consistent 18S fluorescence was observed across fungal hyphae when grown on PD media compared to ME, which featured hyphae with low or no intensity (Figure 3). Fungi grown on VM and R2A media exhibited the highest intensity and consistency of 18S labeling and the highest intensity for 16S labeling. However, VM-grown fungi were associated with confounding autofluorescence making bacterial analysis more difficult. Wide field of view images are provided in Supplementary Figure 4.

In order to assess the impact of culture media composition on endobacterial density, bacteria labeled for 16S rRNA were counted in five images collected across the fungal mycelia for each condition. Because we measured the same fungal samples at the same timepoint with only media being altered, we determined that five images per sample in this analysis would provide a relatively strong representation of the effects from each media type of the data set. From this bacterial quantitation within *P. verticillata* was found an average of 20 and 30 cells per 100 square micron area for the PD and R2A samples, while the ME samples were found to have harbored relatively fewer bacteria, with less than 5 cells per 100 square micron area (w/PD *p* = 0.007, w/R2A *p* < 0.001). VM was not included in the measurements due to high levels of autofluorescence. The variability observed in this experiment suggests that culture media composition can impact interpretations of endobacterial prevalence within fungal isolates. Furthermore, fluorescence-based imaging studies of diverse BFI may require unique optimizations or considerations with respect to culture media composition based on the fungal host, the type of association, and the bacterial associate.

### Uneven Distribution of Endobacteria Across Fungal Mycelia

**Figure 4:**
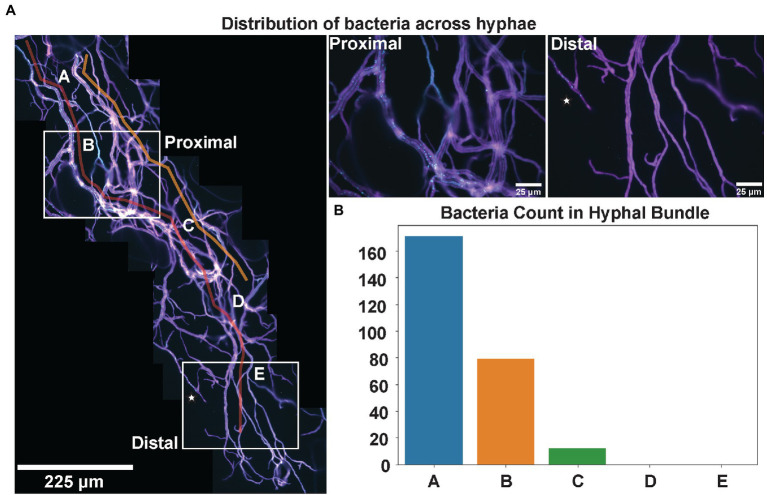
**(A)** *Mycoavidus* (Cyan) observed across the length of *P. verticillata* (LANL.1351.123) hyphal bundles (Magenta) extending from the site of inoculation (Proximal) to the nascent hyphal tips away from the inoculation site (Distal). Guide lines highlighted in Red and Orange indicate hyphae of interest exhibiting high bacterial coincidence (Red) and low bacterial incidence (Orange). Hypha denoted by star in Distal end indicates a nascent strand that has greater accessibility for *Mycoavidus* to travel toward the tip. Higher magnification images of locations of the Proximal and Distal ends are provided on the right. **(B)** *Mycoavidus* count from several images throughout the hyphal bundle (denoted A–E) outlined in Red showing decreasing bacterial observations toward the hyphal tips.

An additional challenge to performing microscopic measurements within filamentous fungi, as opposed to unicellular microbes, such as yeast, is determining the regions of interest for microscopy from a heterogeneous system (Zacchetti et al., 2018). This investigation demonstrated that variable bacterial numbers could be observed depending on the media, the field of view, or area of the fungal culture being examined. The highest density of bacteria was observed within the oldest hyphae immediately extending from the inoculation site (Supplementary Figure 5). We then investigated how the density of endobacteria changed across entire fungal hyphal strands. In Figure 4A, a stitched image of *P. verticillata* hyphae shows the spatial distribution of *Mycoavidus* extending from proximal regions to the inoculation point to more distal nascent hyphal tips. Interestingly, decreasing numbers of bacteria were observed toward nascent hyphal tips (red guiding line, Figure 4A). The accompanying bar plot also shows decreasing bacterial counts across several locations along the hyphae as it extends toward the hyphal tip (Figure 4B). Further emphasizing the spatial heterogeneity, the bacterial occupancy is hyphal strand dependent as some hyphae lacked endobacterial symbionts, which was observed in the hyphal bundles adjacent to the orange guiding line (Figure 4A).

### Enhancement of Endohyphal Bacterial Staining

**Figure 5:**
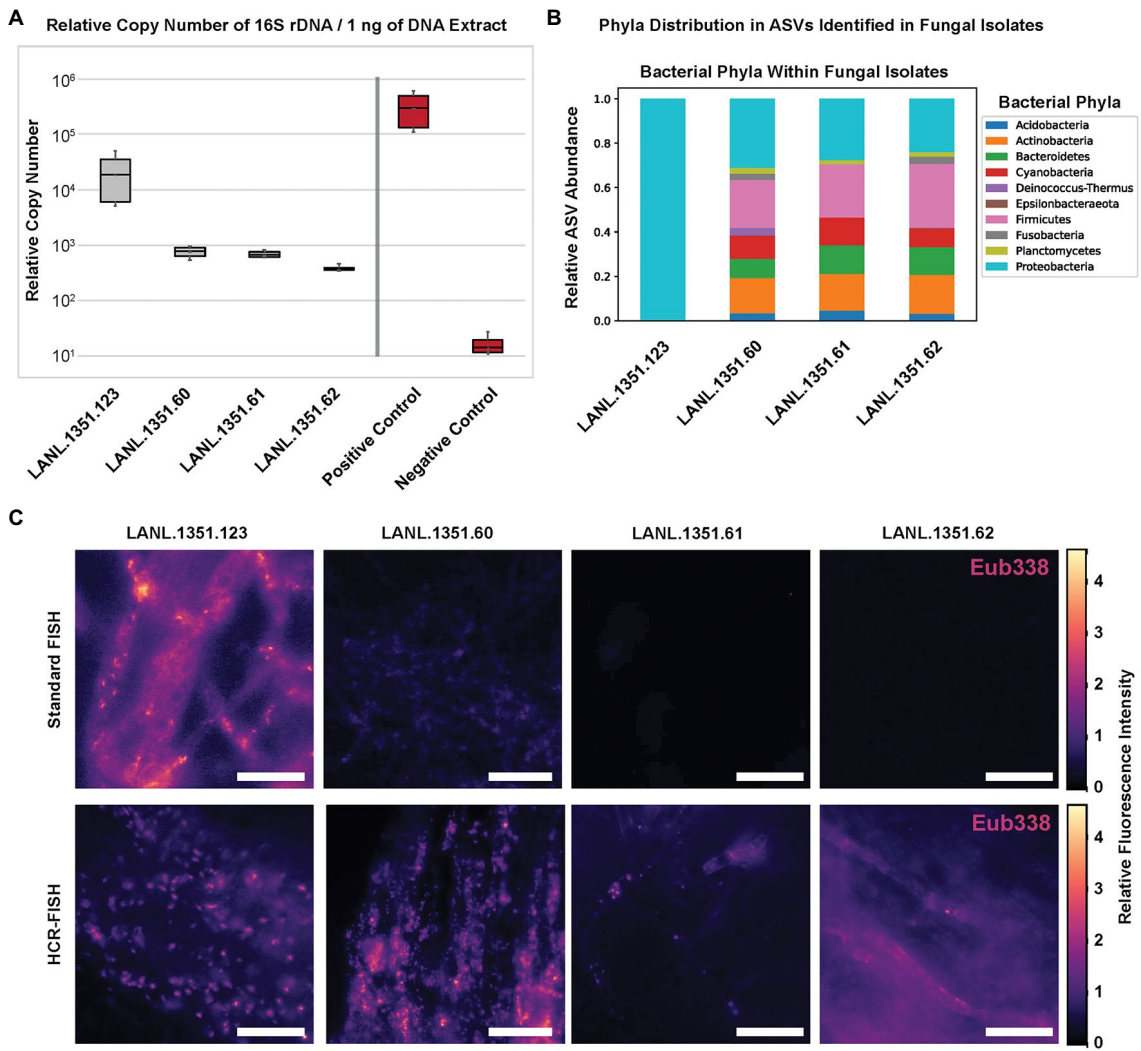
**(A)** Relative copy number of bacterial 16S rDNA amplified from total DNA extraction from three replicates of one *P. verticillata* isolate, three *M. alpina* isolates (LANL.1351.123, LANL.1351.60, LANL.1351.61, and LANL.1351.60) and two control *Rhizopus* isolates and quantified against a calibration curve of 16S rRNA gene from a *Burkholderia* standard. **(B)** Bacterial phyla relative read count abundance from 16S ASVs acquired from MiSeq sequencing of DNA from the four Mortierellaceae isolates. Read counts are normalized to 1. *Mycoavidus* represents the Proteobacteria for LANL.1351.123 exclusively. **(C)** Relative fluorescence images of *Mycoavidus* bacteria (scaled to color map) observed in mycelial dissections from the four Mortierellaceae isolates stained either by standard 16S rRNA FISH (Standard FISH; top) or Hybridization Chain Reaction (HCR-FISH; bottom) methods. The fluorescence intensities are scaled to the minimum and maximum pixel intensities across images of each row. Scale bar is 25 μm.

The above bacterial imaging with FISH under variable conditions was examined using *P. verticillata*, LANL.1351.123, primarily due to the high abundance of *Mycoavidus* in the fungal mycelia examined by qPCR quantitative analysis of 16S rRNA gene copy numbers (Figure 5A). While *Mycoavidus* has been the focus of many endosymbiont investigations, recent work by Robinson et al. indicated that other isolates from Mortierellaceae are capable of harboring diverse bacteria (Robinson et al., 2021), many of which appear to be less prevalent than what is typically observed in associations with *Mycoavidus* (Figures 5A,B). To investigate these less abundant bacterial associates within our isolates, we tested endobacterial staining procedures to assess our ability to detect and visualize bacterial associates at orders of magnitude lower than LANL.1351.123 (as determined by 16S rDNA qPCR quantitation and 16S amplicon sequencing) including three *Mortierella alpina* isolates (LANL.1351.60, LANL.1351.61, and LANL.1351.62) lacking *Mycoavidus* under the Proteobacteria phylum (Supplementary Figure 6).

In order to increase the fluorescence signal and improve the SNR, hybridization chain reaction FISH (HCR-FISH) was used to amplify the 16S rRNA signal based on the Eub338 sequence. HCR-FISH is a signal amplification technique that employs extended hybridization probes that include both a target recognition sequence and an initiator sequence. When the initiator is recognized by the amplification probes composed of two fluorescently labeled kinetically trapped hairpins, it drives the opening and binding of the probes to the initiator and each other to generate a growing double-stranded polymer of alternating emitters (Dirks and Pierce, 2004; Bi et al., 2017; Choi et al., 2018). Under standard fluorophore labeling with the universal 16S probe, Eub338, fluorescence showed little to no bacterial labeling in several images of fungal isolates outside the LANL.1351.123 isolate harboring *Mycoavidus* (representative images are shown in Figure 5C, top). However, upon amplification of the Eub338 HCR-FISH probe, distinct fluorescent features were observed in images taken across the fungal hyphae relating to the labeled bacterial 16S rRNA (representative images are shown in Figure 5C, bottom). Additional higher magnification images confirm distinct objects and morphologies in the *M. alpina* isolates that do not have *Mycoavidus* bacteria (Supplementary Figure 7). Fluorescent staining by HCR confirms the presence of bacteria in low density within several *M. alpina* isolates as detected with qPCR, albeit also requiring amplification to detect.

## Discussion

In this study, a number of growth conditions and physical and chemical treatments were examined to understand their impact on imaging bacteria within the mycelia of filamentous fungi. We note that imaging *via* microscopy can easily be obfuscated by a multitude of factors, such as the thickness of the mycelial mat, location of bacteria across the fungal hyphae, the concentration of targets investigated, and even the unique biology of particular fungal taxa. As the field of BFI research continues to expand to include broader investigations of diverse associations, it is important to consider how differences in sample preparation and imaging methodologies could alter interpretation or comparisons based on imaging. Standardization of microscopic investigations and techniques, both for BFI and more generally for mycological and microbiological research, will be essential to leveraging images as a datasource to complement sequence-based and qPCR approaches.

One of the most dramatic improvements observed in mycelial imaging was the use of thin hydrogel substrates for growing fungal cultures. Using hydrogel substrates for growing and imaging the fungi made it possible to clearly distinguish individual hyphae and their associated bacteria, suggesting that preparation aids in preserving the native fungal structures similarly observed in previous reports (Woo et al., 2010; Pampaloni, 2017). For *P. verticillata*, this also permitted acquisition of informative images with little obfuscation due to thick mycelial mats that are often unavoidable when making dissections from fungi grown on Petri dishes. Media discs also promoted adherence of fungi to the surface minimizing movement for performing confocal microscopy 3D images and across lateral distances. We also observed that depending on the hydrogel used, we could minimize autofluorescence conferred by the substrate itself. Consistent with the findings from Jaeger et al. (2015), Phytagel provided the best clarity for fluorescence microscopy. Furthermore, the hydrogel discs are not only amenable to slides fitted with reagent barriers but also for culturing within 12- and 24-well plate format. There are few methods available to perform high-throughput image-based screening for fungi (Golabgir et al., 2015; Beneyton et al., 2016), and even fewer options suitable for methods like FISH. By reducing the three-dimensional footprint of the substrate, we are able to reduce the volume of reagents necessary for staining and open the opportunity for parallelization and higher sample throughput. Increased sample throughput will enable expanded investigations into the diversity of BFI across different environments and facilitate comparisons between established BFI models.

Based on our observations in *P. verticillata*, chemical and enzymatic treatments that diminish the integrity of the fungal cell wall have potential benefits for improving the efficiency and quality of fluorescence imaging with FISH. Intriguingly, moderate labeling of intracellular bacterial and fungal rRNA was observed without the treatment of either chemical or enzymatic digestion of the cell wall in the examined fungal isolate. This could be owed to the partial permeabilization of cell membranes by fixation. As cells are exposed to the hypertonic solution during formaldehyde fixation, the membrane integrity deteriorates in response to osmotic stress which potentially permits molecules <60 kDa intracellular access (Cheng et al., 2019; Flores Bueso et al., 2020). The penetration of probes into the fungus, however, was dramatically improved upon enzymatic and chemical treatment. With the addition of just lysozyme, significant autofluorescence was observed across all fluorescence channels. Previous studies have shown that lysozyme can non-specifically bind to DNA or RNA by electrostatic interactions and could promote non-specific association of the fluorescent probes to the fungi (Ghosh et al., 2013). Combining lysozyme treatment with glucanase and/or chitinase, on the other hand, improved SNR. This was further enhanced with the addition of ethanol, which is consistent with the fact that ethanol solubilizes cellular membrane components and dehydration establishes a greater osmotic gradient to draw more probes into cells (Haroon et al., 2013; Rocha et al., 2018). Since fungal cell wall composition can be variable across the fungal tree of life, it would be valuable to evaluate the effect of similar treatments in diverse fungal isolates toward standardization to enable increased comparisons of imaging data. While ultimately custom digestion and permeabilization may be necessary to find the best combination of conditions suitable for the fungus and experiment of interest, this work provides a foundation from which to begin exploring these questions.

In studying endobacteria in fungi, some consideration should be placed on the conditions of the media to optimize the growth of both organisms. In soil, both fungal and bacterial growth are influenced by a multitude of conditions, such as pH, and they can compete for the same resources and impact the others’ growth rate (Vieira and Nahas, 2005; Rousk et al., 2010; Black, 2020). We tested several culturing media to determine if the number of bacteria changed with altered conditions. In the cultures we prepared, we found that *Podila* grown on PD and R2A media yielded similar bacterial counts, but the number of bacteria observed in fungi grown on ME was approximately 5-fold lower. This agrees in part with previous reports that the acidic pH of ME (pH ~4.7) favors the growth of fungi while restricting bacterial growth (Jarvis, 1973) compared to PD and R2A (pH ~7). In contrast, media may not be responsible for bacterial growth as it has been observed that the pH of the intracellular region of *Aspergillus niger*, remains approximately neutral when grown in either acidic or basic conditions (Bagar et al., 2009) and instead this could be due to the lack of dextrose and starch in ME. Additionally, *Mycoavidus cysteinexigens* sp. nov, the endosymbiont of *Mortierella elongata*, along with several strains of bacteria from the family Burkholderiaceae were reported to be unable to utilize maltose (Zhang et al., 2000; Ohshima et al., 2016) suggesting the *Mycoavidus* examined here may be metabolically restricted and reliant on host metabolism, thus slowing growth. Vogel’s media in this study was associated with increased autofluorescence making automated quantitation difficult, but qualitatively still exhibited endobacterial growth.

Fungal mycelia are heterogeneous across hyphae, and we observed variable distribution of *Mycoavidus* across fungal mycelia. Using image reconstruction of extended hyphal networks from a series of high magnification images, we can capture spatiotemporal dynamics of filamentous fungi (Dikec et al., 2020). *Podila verticillata* used here exhibits predominantly non-septated mycelia and no obvious divisions between fungal cells. We determined that the bacterial presence across fungal hyphae was dynamic and exhibited greater bacterial counts when imaged closer to the inoculation site of the fungi while there were minimal bacteria found at the nascent hyphal tips. Furthermore, bacterial numbers were variable between hyphae bundles and some hyphae had considerable quantities of bacteria while others had none. These observations could suggest that there is independent division and movement of bacteria across the fungus. Phenotypic heterogeneity of a broad range of filamentous fungal phenotypes has been well established (Roper et al., 2011; Hewitt et al., 2016). However, there is an interconnectedness, or syncytia, in hyphal architecture that is suggested to link individual behavior like organelle motility (e.g., nuclei and mitochondria) to the colony as a whole (Mela et al., 2020). The inclusion of seemingly autonomous bacteria adds an additional layer of complexity to fungal heterogeneity which brings into question how these relationships are governed. Extending these studies toward septated fungi will be quite interesting to understand the developmental cues for bacteria and spatial distribution of the bacteria across single fungal cells.

The use of microscopy to visualize and identify endobacteria in fungi is a robust strategy to validate bacterial presence and location within the mycelia. However, as reported by Robinson et al. these BFI are beginning to be understood as quite cosmopolitan and do not always present the same fluorescence intensity as observed with *Mycoavidus* in *P. verticillata* (Robinson et al., 2021). 16S rRNA gene amplicon sequencing and qPCR provide ensemble evidence that bacteria are associated with certain fungal isolates. However, visualizing these symbionts is also necessary. Visualizing with standard probe labeling is challenging either due to low bacterial abundance or low 16S target transcript abundance. Previous reports have also found standard FISH imaging insufficient for capturing targets with low ribosomal RNA content and have suggested amplification techniques like HCR-FISH to be quite valuable in ecological environmental samples and visualizing symbiotic associations (Nikolakakis et al., 2015; Yamaguchi et al., 2015; Imachi et al., 2020). We showed that using HCR-FISH to amplify the signal improves the SNR and provides a greater level of confidence that there are bacterial and fungal associations present. This visual confirmation of physical associations helps validate and complements previous sequence-based discovery of novel bacterial–fungal associations and provides a more solid basis for probing into these poorly defined inter-kingdom relationships. As more BFI are elucidated, it is clear that many of these associations will require investigation by HCR-FISH or other amplification methods to visually confirm and study less-obvious bacterial inhabitants present at low abundance.

## Conclusion

Bacterial–fungal interactions, while ubiquitous, remain largely unexplored. While next-generation sequencing methods have been exploited at a rapid pace to characterize BFI, there is a need for advancement in optical microscopy methods to verify and visualize these BFI across space and time. Here, we examined a single model system of *P. verticillata* and explored the aspects that influence fungal growth and preparation for consistently visualizing endobacteria. Going forward, *in situ* techniques, such as FISH, provide the clearest spatial and temporal view of how these microbes associate. However, to reach beyond Mucoromycota fungi, several considerations should be made in investigating bacterial interactions with fungal mycelia by microscopy. Digestion of fungal cell walls and achieving membrane permeability, optimizing growth conditions on optically clear substrates, and establishing spatial context will require custom procedures to fully interrogate these systems.

## Data Availability Statement

The datasets presented in this study can be found in online repositories, further inquiries can be directed to the corresponding author. The names of the repository/repositories and accession number(s) can be found at: https://www.ncbi.nlm.nih.gov/genbank/, MZ374644.1 https://www.ncbi.nlm.nih.gov/genbank/, MZ374682.1 https://www.ncbi.nlm.nih.gov/genbank/, MZ374683.1 https://www.ncbi.nlm.nih.gov/genbank/, MZ374684.1 https://www.ncbi.nlm.nih.gov/, SAMN19716290 https://www.ncbi.nlm.nih.gov/, SAMN19716328 https://www.ncbi.nlm.nih.gov/, SAMN19716329 https://www.ncbi.nlm.nih.gov/, SAMN19716330 https://www.ncbi.nlm.nih.gov/genbank/, JALPZK000000000 https://www.ncbi.nlm.nih.gov/, ON357719 https://www.ncbi.nlm.nih.gov/, ON357872.

## Author Contributions

DM contributed to the planning, experimentation, analysis, data interpretation, and writing of the enclosed manuscript. AR contributed to the planning, experimentation, data interpretation, and writing of the manuscript. AP contributed to the method and probe design. CA contributed to the probe design. JK contributed to the planning and writing of the manuscript. PJ contributed to the planning and interpretation. JW and PC contributed to the planning, data interpretation, and writing of the manuscript. All authors contributed to the article and approved the submitted version.

## Funding

This study was supported by the U.S. Department of Energy, Office of Science, Biological and Environmental Research Division, under award number LANLF59T.

## Conflict of Interest

The authors declare that the research was conducted in the absence of any commercial or financial relationships that could be construed as a potential conflict of interest.

## Publisher’s Note

All claims expressed in this article are solely those of the authors and do not necessarily represent those of their affiliated organizations, or those of the publisher, the editors and the reviewers. Any product that may be evaluated in this article, or claim that may be made by its manufacturer, is not guaranteed or endorsed by the publisher.

## Footnotes

^1^ https://doi.org/10.1186/s12859-014-0366-2

^2^ https://doi.org/10.1093/nar/gkw1027

^3^ https://doi.org/10.1002/cpbi.102

^4^ https://github.com/tseemann/barrnap

^5^ https://blast.ncbi.nlm.nih.gov/Blast.cgi

## Supplementary Material

The Supplementary Material for this article can be found online at: https://www.frontiersin.org/articles/10.3389/fmicb. 2022.892227/full#supplementary-material
