# Supplementary material for: Advances and Challenges in Fluorescence in situ Hybridization for Visualizing Fungal Endobacteria
Source: Front Microbiol. 2022 May 26;13:892227. doi: 10.3389/fmicb.2022.892227 (PMC9199388; doi:10.3389/fmicb.2022.892227)
Supplement: Supplementary file 1 [file Data_Sheet_1.pdf]

Supplementary Material

Supplementary Figures

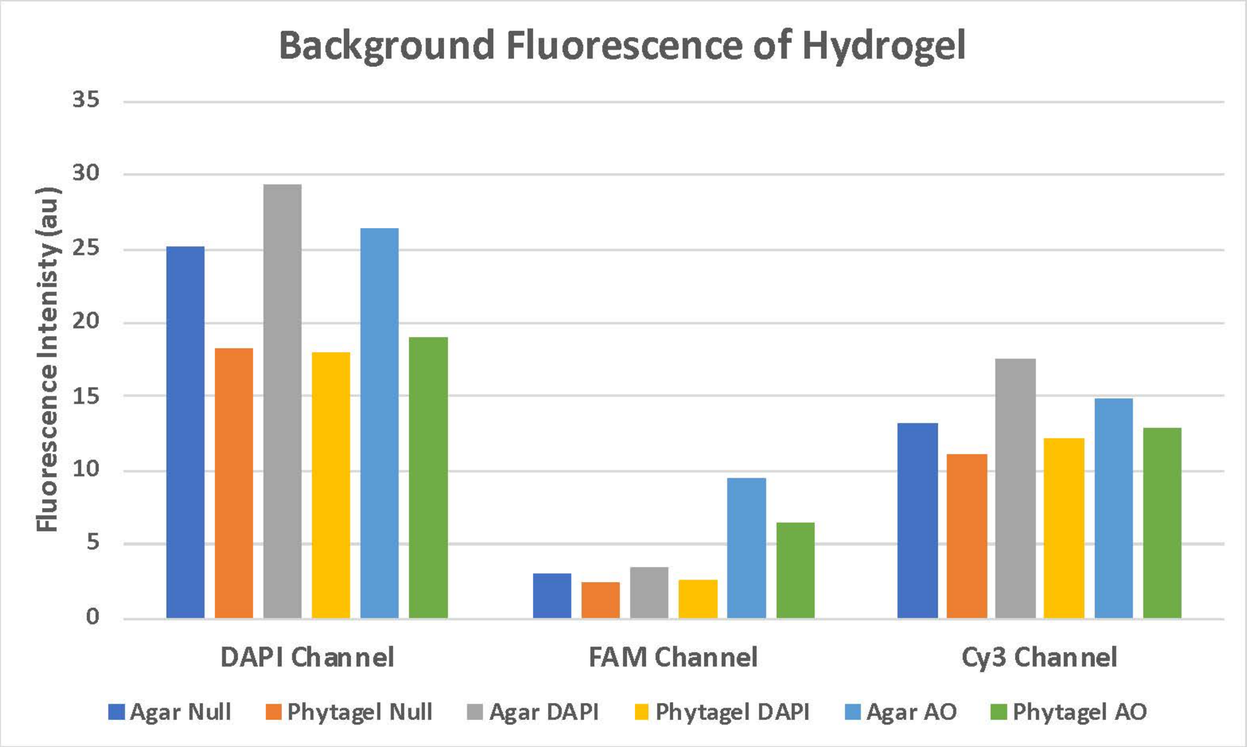

Supplementary Figure 1: Mean pixel autofluorescence/background fluorescence observed in hydrogels relative to buffer of 2X SSC alone on coverslip. Three fluorescence channels were tested (DAPI, FAM, and Cy3) for hydrogels stained with buffer, a working solution of 1 µg/mL DAPI, and a working solution of 10 µg/mL acridine orange. Phytagel exhibited the least auto-/background fluorescence of the two hydrogels across the fluorescence channels and staining treatments.

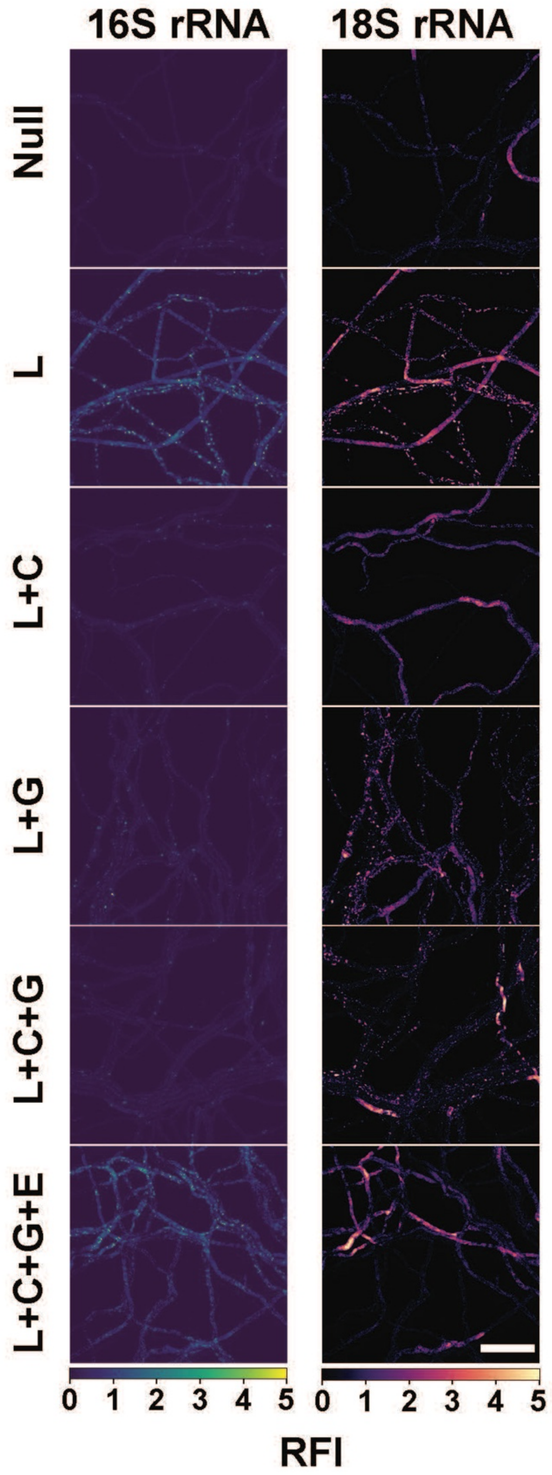

Supplementary Figure 2: Widefield images of *Mycoavidus* (16S rRNA) observed in *P. verticillata* (18S rRNA) exposed to different chemical or digestion treatments for cell wall permeabilization. Relative fluorescence intensity images are scaled to the minimum and maximum pixel intensities of image sets (top to bottom) for 16S rRNA or 18S rRNA staining. Single letter code: L = Lysozyme; C = Chitinase; G = Glucanase; E = Ethanol. Scale bar is 25 µm.

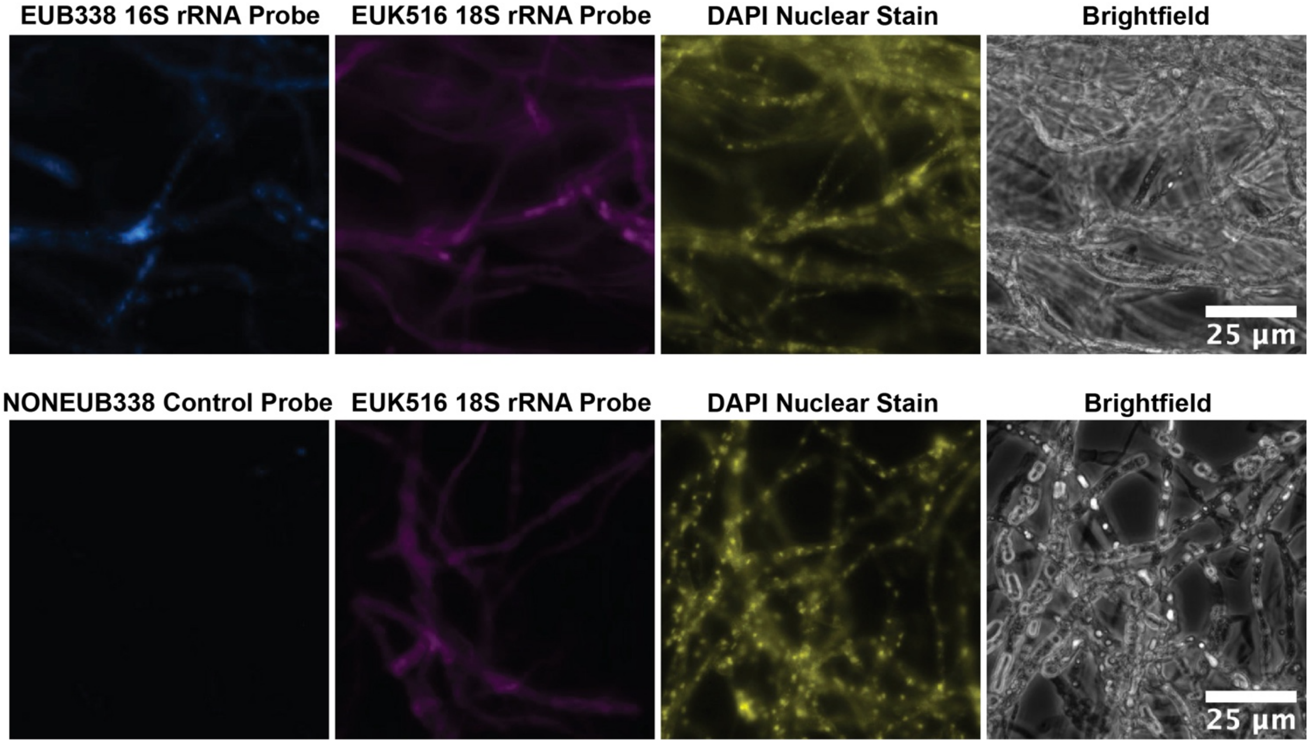

Supplementary Figure 3: Generalist probe staining of *P. verticillata* stained with DAPI and Euk516 (Amann et al., 1990) 5'- ACC AGA CTT GCC CTC C -3' and *Mycoavidus* stained with EUB338 (Amann et al., 1990) 5'- GCT GCC TCC CGT AGG AGT -3' and NONEUB338 (Wallner et al., 1993) 5'- ACT CCT ACG GGA GGC AGC -3'.

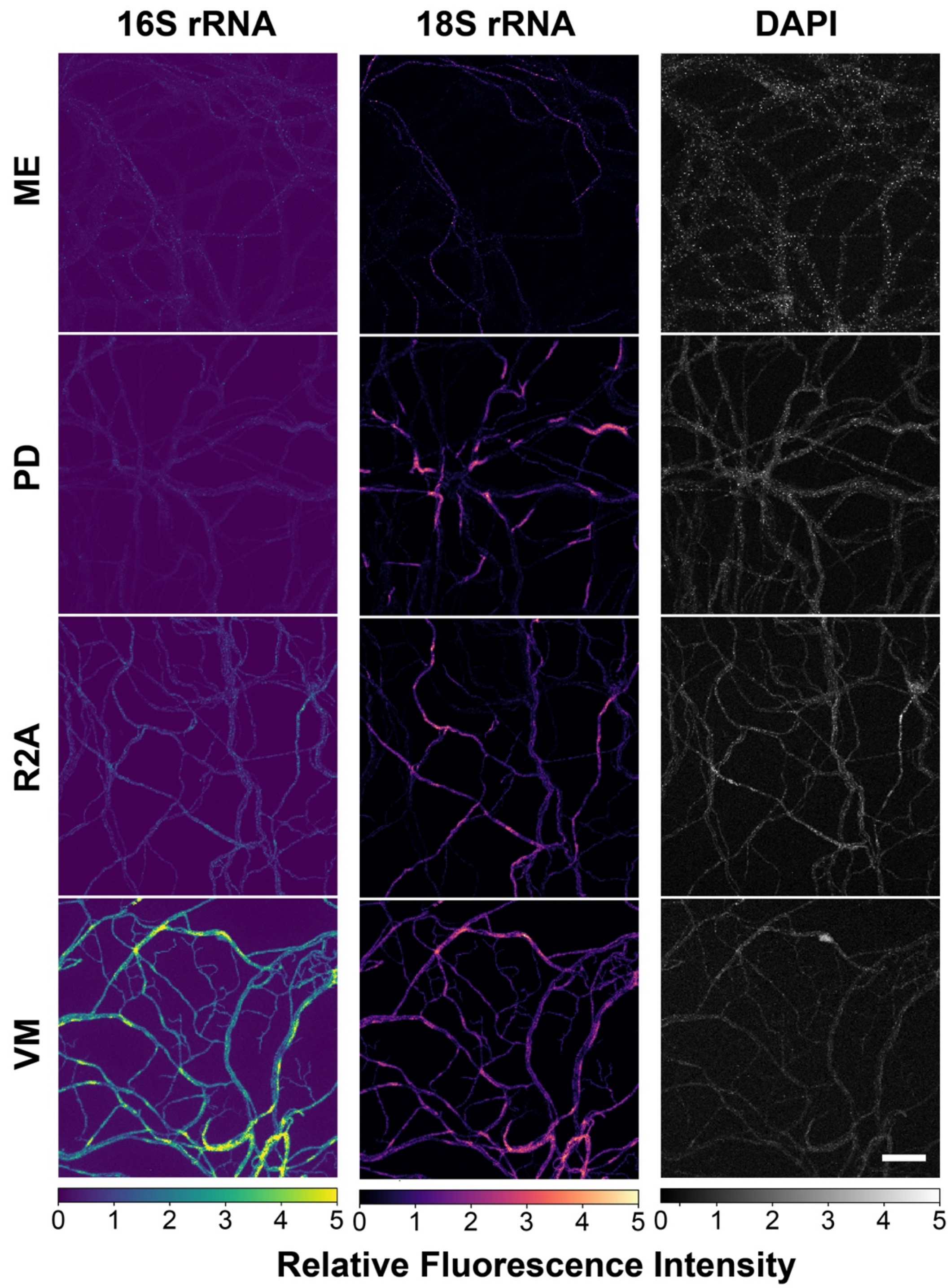

Supplementary Figure 4: Widefield images of *Mycoavdius* (16S rRNA) observed in *P. verticillata* (18S rRNA) grown on Phytagel hydrogel discs supplemented with either Malt Extract (ME), Potato Dextrose (PD), Reasoner's 2A (R2A), Vogel's minimal media (VM). Relative fluorescence intensity images are scaled to the minimum and maximum pixel intensities of image sets (top to bottom) for 16S rRNA, 18S rRNA, or DAPI (nuclear) staining. Scale bar is 50 μm.

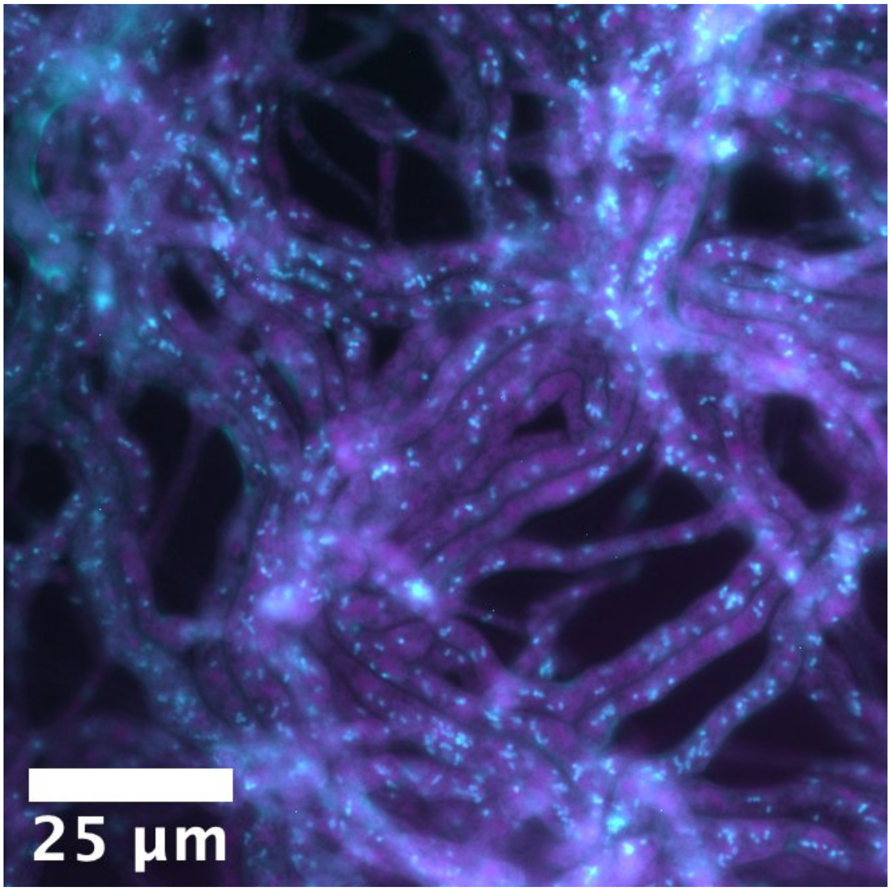

Supplementary Figure 5: Characteristic image of *P. verticillata* (Magenta) immediately extending from the inoculation site exhibiting high concentrations of *Mycoavidus* (Cyan) within the hyphae.

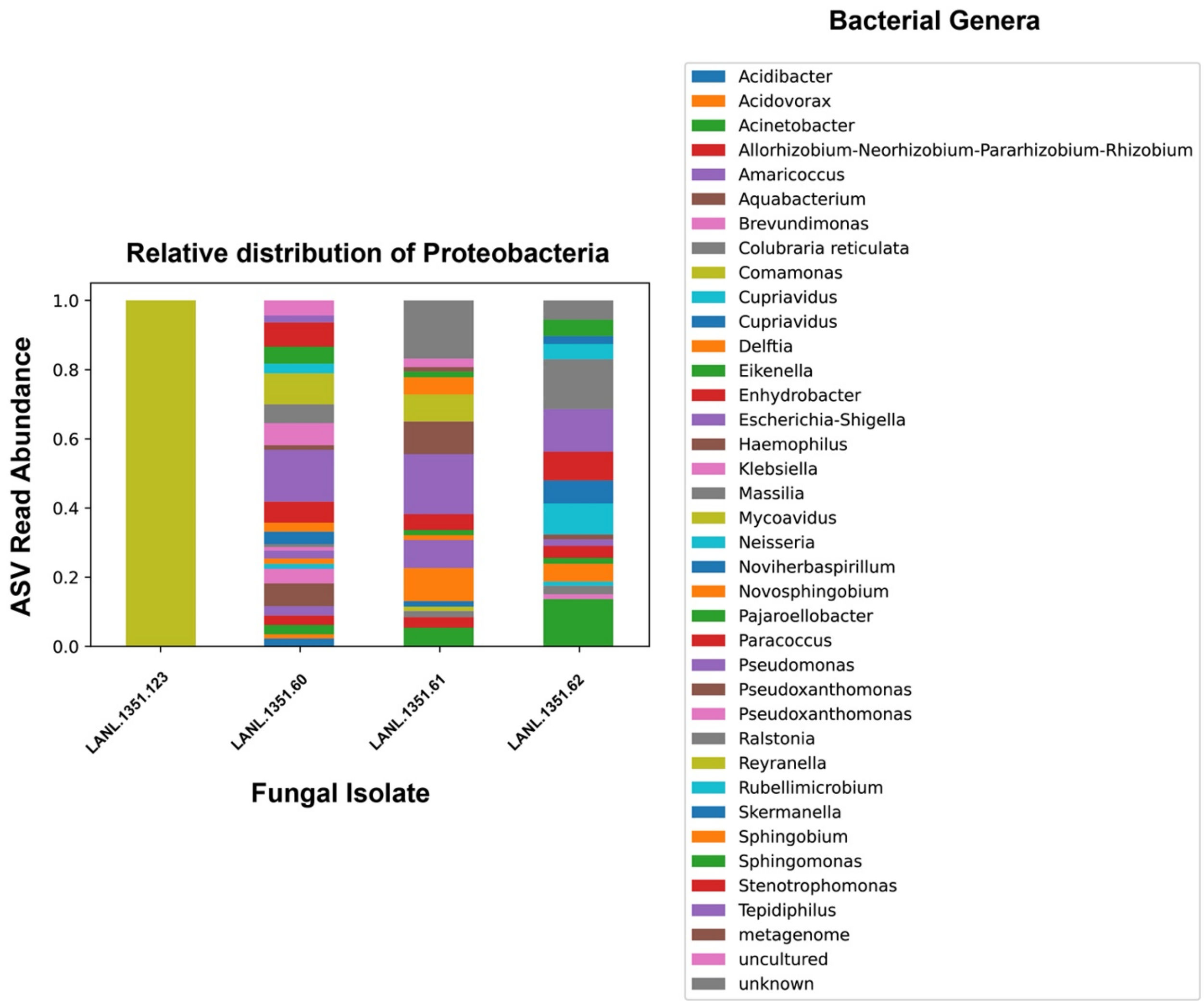

Supplementary Figure 6: Relative abundance of genera diversity within Proteobacteria found in Mortierellaceae isolates. LANL.1351.123 is exclusively *Mycoavidus*, whereas other isolates lack the *Mycoavidus* bacterium.

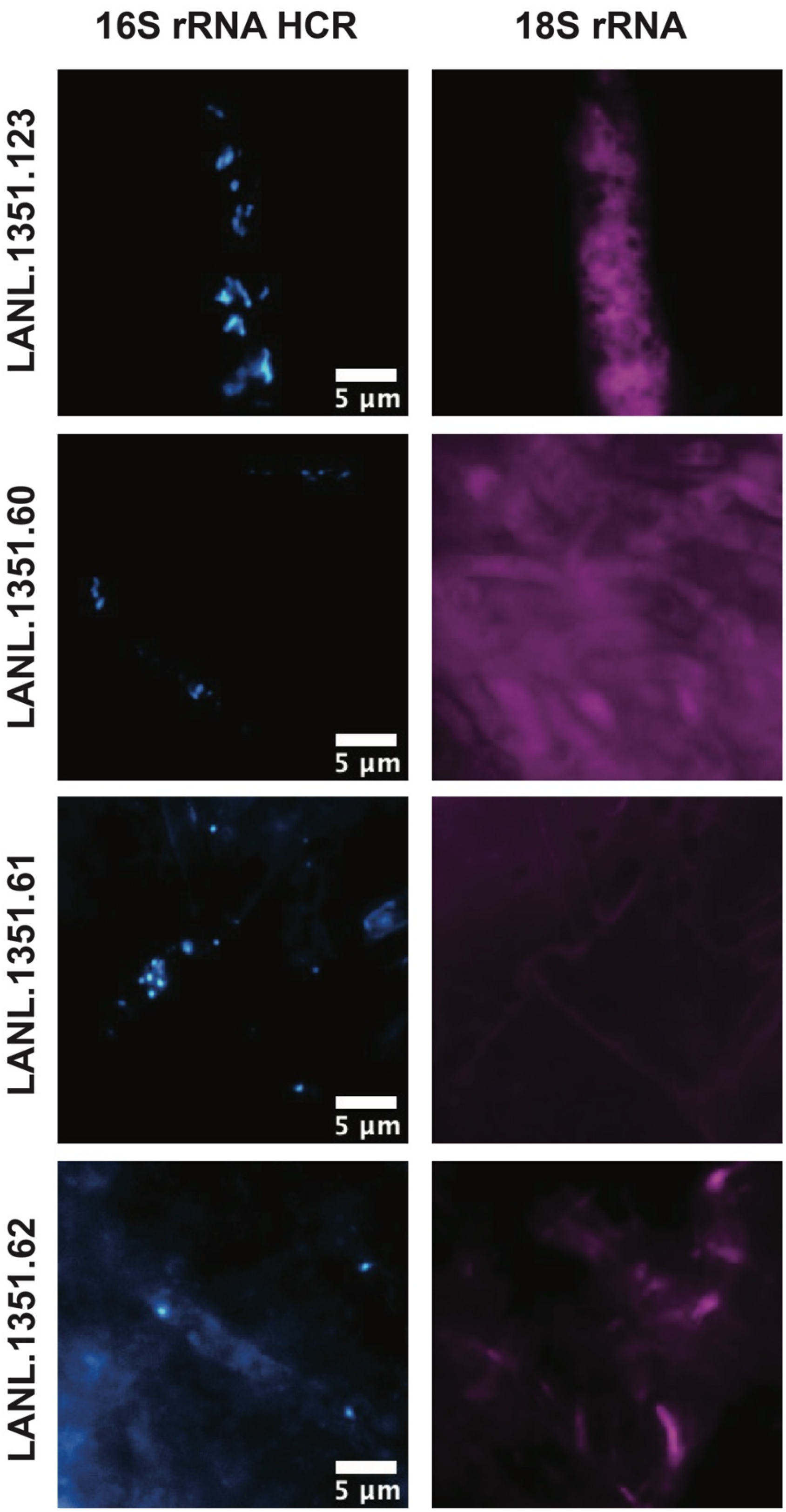

Supplementary Figure 7: High magnification images of bacteria (universal 16S probe) observed in several Mortierellaceae isolates after hybridization chain reaction FISH. A logarithmic color space scale was used for lookup table representation for the 16S for clarity.

References

Amann, R.I., Binder, B.J., Olson, R.J., Chisholm, S.W., Devereux, R., and Stahl, D.A. (1990). Combination of 16S rRNA-targeted oligonucleotide probes with flow cytometry for analyzing mixed microbial populations. *Applied and Environmental Microbiology* 56(6)**,** 1919-1925. doi: doi:10.1128/aem.56.6.1919-1925.1990.

Wallner, G., Amann, R., and Beisker, W. (1993). Optimizing fluorescent in situ hybridization with rRNA-targeted oligonucleotide probes for flow cytometric identification of microorganisms. *Cytometry* 14(2)**,** 136-143. doi: 10.1002/cyto.990140205.
